# Supplementary material for: Shape optimization for high efficiency metasurfaces: theory and implementation
Source: Light Sci Appl. 2024 Oct 29;13:300. doi: 10.1038/s41377-024-01629-5 (PMC11519467; doi:10.1038/s41377-024-01629-5)
Supplement: Supplementary file 1 — Supplementary information for Shape optimization for high efficiency metasurfaces: theory and implementation [file 41377_2024_1629_MOESM1_ESM.pdf]

Supplementary information for

Shape optimization for high efficiency metasurfaces: theory and implementation

Paulo Dainese[1], Louis Marra[1], Davide Cassara[2], Ary Portes[2], Jaewon Oh[2], Jun Yang[1], Alfonso Palmieri[2], Janderson Rocha Rodrigues[1], Ahmed H. Dorrah[2], and Federico Capasso[2]

[1]*Corning Research and Development Corporation, 184 Science Center Dr, Painted Post, NY 14870, USA*

[2]*Harvard John A. Paulson School of Engineering and Applied Sciences 1, Cambridge, MA 02138, USA*

1 Mathematical formulation

The adjoint formulation is discussed in 1.1, including the definition of the boundary gradient. A validation of the gradient with two numerical examples is provided in 1.2, and the Fourier decomposition is discussed in 1.3. Finally, a generalization to an arbitrary figure of merit is discussed in 1.4.

1.1 Adjoint-based boundary gradient

The objective when designing a metasurface is to determine a specific configuration in the constituent materials, meaning their geometrical shapes and dielectric permittivity, so that an incoming field is transformed into a target field E_t at the output surface S (Figure S1a). Suppose we start with an arbitrary configuration represented by a dielectric permittivity distribution ϵ, which in general varies in space, and then change it from $\epsilon' = \epsilon + \delta\epsilon$ by modifying a certain region χ within the optimization domain (Figure S1b). For example, ϵ may represent an arrangement of meta-atoms (with permittivity ϵ_2) embedded in a background material (with permittivity ϵ_1). Enlarging one of the meta-atoms changes the permittivity in the region χ from the background value ϵ_1 to the meta-atom's permittivity ϵ_2. In general, a set of perturbations create a new configuration represented by ϵ'. The question we want to answer is where and how to change the structure so that the field changes from E to $E' = E + \delta E$, moving in a direction closer to our target field E_t at the output surface S.

Typically, a figure of merit is used to 'measure' how close the field E is to the target E_t. For example, E_t might be the mode of a waveguide positioned at the output of the domain or simply a desired field in free-space (such as a particular diffraction order in a grating or a focused Gaussian beam and so on). We can express the projection of the field E into the target field E_t as:

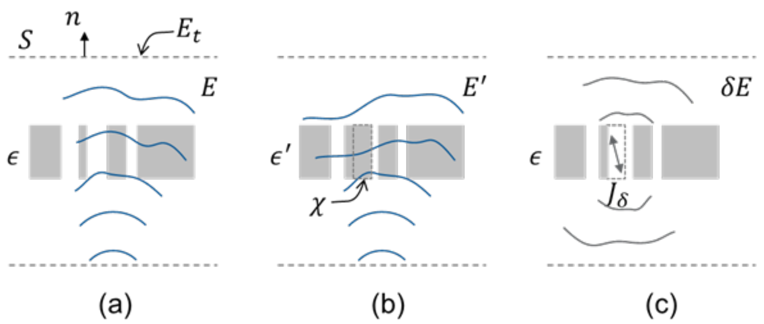

Figure S1: (a) an incident field interacts with a material configuration with permittivity ϵ to generate a field E in space; (b) a new configuration $\epsilon' = \epsilon + \delta\epsilon$ generates a field $E' = E + \delta E$ (note that the region where the permittivity changed is denoted by χ). In (c), the same field correction δE can be produced in the initial configuration ϵ when a proper current J_δ is added in the perturbed region χ.

$$F = \frac{1}{4\,P} \int_S \left(E \times H_{\mathrm{t}}^* + E_{\mathrm{t}}^* \times H\right) \cdot n da \tag{S1}$$

where n is the normal unit vector at the output surface S, pointing outwards from the simulation domain. If we normalize both the incoming and target fields (P=1 W), then the efficiency of our device is directly obtained by $\eta = |F|^2$. Any modification in the metasurface structure that changes the fields by $(\delta E, \delta H)$ impacts the efficiency as:

$$\delta\eta = 2\,\mathrm{Re}\left[F^* \delta F\right] \tag{S2}$$

where

$$\begin{aligned}\delta F &= \frac{1}{4\,P} \int_S \left(\delta E \times H_{\mathrm{t}}^* + E_{\mathrm{t}}^* \times \delta H\right) \cdot n da \\ &= \frac{1}{4\,P} \int_S \left[-\left(n \times H_{\mathrm{t}}^*\right) \cdot \delta E + \left(n \times E_{\mathrm{t}}^*\right) \cdot \delta H\right] da\end{aligned} \tag{S3}$$

The challenge is to determine how to modify the metasurface geometry in each iteration so $\delta\eta$ increases, as opposed to brute force trial and error every possible modification. The derivation goes as follows: we first perform two simulations to extract information in the existing medium configuration, so called forward and adjoint simulations. In the forward simulation, we calculate the field E for the unperturbed medium ϵ excited by our input source. This allows us to estimate any new polarization currents J_δ that might be generated when the medium is modified by $\delta\epsilon$ (i.e., any small amount of material placed on the surface that generates a new polarization current), as illustrated in Figure S1c. In the adjoint simulation, we place specific current sources J_{a} on the output surface S that excites our medium backwards, generating a field in our domain denoted by E_{a}. We choose the currents such the adjoint field is equivalent to propagating our target field backwards into the medium. The next step is to relate these currents J_{a} and J_δ using the reciprocity theorem. This will give us a recipe for choosing where to place J_δ (i.e. where to place $\delta\epsilon$) that ensures our efficiency increases. We now follow these steps mathematically.

The field correction δE obtained in the perturbed medium ϵ' can be generated in the 'known' configuration ϵ if a proper current J_δ is placed in the perturbed regions χ, where:

$$J_\delta = j\omega\delta\epsilon\, E' \tag{S4}$$

This is illustrated in Figure S1c. Physically, this current is the time derivative of the additional polarization in the medium $J_\delta = \partial_t \delta P = \partial_t \delta\epsilon E' = j\omega\delta\epsilon E'$, where we assume all fields are harmonics with dependency $e^{j\omega t}$. In

other words, J_δ are the polarization currents in the medium. This can be seen by directly writing down Ampère's law in a source free region in both perturbed and unperturbed medium:

$$\nabla \times \ H = j\omega\epsilon \, E \quad \text{and} \quad \nabla \times \ H' = j\omega\epsilon' E' \tag{S5}$$

where again $\epsilon' = \epsilon + \delta\epsilon$ and $E' = E + \delta E$. Subtracting one from the other we obtain directly:

$$\nabla \times \delta \, H = j\omega\epsilon\delta \, E + J_\delta \tag{S6}$$

The last equation states that the fields $(\delta E, \delta H)$ are generated in the unperturbed medium ϵ by the current source J_δ. At first this expression does not seem to be very useful because we do not yet know the perturbed field E' (and therefore we can't determine $J_\delta = j\omega\delta\epsilon E'$). However, note that we do not need to know E' everywhere in space, we only need to find an approximation for E' in the perturbation regions χ, i.e., where $\delta\epsilon$ is non-zero. This is illustrated in Figure S2. The dashed line represents the original boundary of a given feature (a circular pillar in this case) that is then distorted by an amount $u_\perp$, which of course can vary along the perimeter of the shape. The tangential component of the electric field is continuous across the boundary and therefore can be considered approximately unchanged if the boundary displacement is infinitesimal (i.e. $E'_{\|} = E_{\|}$ for $u_\perp \to 0$). This is however not true for the normal electric field. Since it is discontinuous, the field change is finite no matter how small the boundary displacement is [1]. We can circumvent this problem by noting that the normal displacement field $D_\perp$ is continuous, and therefore:

$$E' \cong \ E_{\|} + \frac{1}{\epsilon'} D_\perp \tag{S7}$$

and so

$$J_\delta \cong \ j\omega\delta\epsilon \left(E_{\|} + \frac{1}{\epsilon\prime} D_\perp \right) \tag{S8}$$

Note that this approximation treats correctly abrupt material boundaries, i.e., with discontinuous permittivity across the boundary [1]. In most topological optimization algorithms, the medium is represented by an approximate continuous permittivity, in which case the field is always continuous and therefore $E' \cong E$. This means that in topology optimization, the material slowly evolves from a continuous distribution, eventually converging to a binary index distribution (i.e. manufacturable medium). This evolution however makes it difficult to control appearances of new boundaries, which can be dealt directly with shape optimization as discontinuous boundaries are naturally

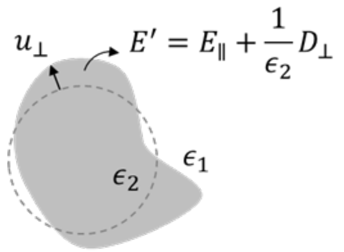

Figure S2: an initial boundary (dashed line) is deformed by a normal displacement field $u_\perp$. In regions where $u_\perp$ is positive the permittivity changes by $\delta\epsilon = \epsilon_2 - \epsilon_1$, while where $u_\perp$ is negative $\delta\epsilon = \epsilon_1 - \epsilon_2$. In the perturbed regions (i.e. near the boundaries), the corrected electric field can be estimated by considering the continuous tangential electric $E_\parallel$ and normal displacement $D_\perp$ fields, obtained in the original configuration.

treated.

For the adjoint simulation, we illuminate the structure with an incident field $(-E_\mathrm{t}^*, H_\mathrm{t}^*)$, where the minus reflects the reversed propagation direction (i.e. propagating into the domain). This can be done through the Principle of Equivalence [2], which states that any incident field into a domain can be produced by equivalent electric and magnetic currents J_a and K_a on the surface S enclosing this domain, where:

$$J_\mathrm{a} = -n \times H_\mathrm{t}^*, \quad \text{and} \quad K_\mathrm{a} = -n \times E_\mathrm{t}^* \tag{S9}$$

where again these surface currents produce a field in our unperturbed domain denoted by E_a. The interpretation of this is clear, it is just the response of the metasurface when excited by our target field instead of our original incident field.

These considerations result in two current-field pairs with a meaningful physical interpretation: the first pair comes from the forward simulation, where we determine that polarization currents J_δ located in the 'to-be-perturbed' regions χ produces the field correction $(\delta E, \delta H)$. The second pair comes from the adjoint simulation, where the currents $(J_\mathrm{a}, K_\mathrm{a})$ located on the domain surface S produce the adjoint field $(E_\mathrm{a}, H_\mathrm{a})$. The goal is now to find a relation between these two pairs that tells us where the currents J_δ should be placed so that $E + \delta E \to E_\mathrm{t}$. This is accomplished by evoking the Lorentz reciprocity theorem [3], which states that in a medium characterized by symmetric (ϵ, μ), the fields (E_1, H_1) and (E_2, H_2), respectively produced by two different sets of localized currents (J_1, K_1) and (J_2, K_2), satisfy the relationship:

$$\int_\Omega (J_1 \cdot E_2 - K_1 \cdot H_2)\, dv = \int_\Omega (J_2 \cdot E_1 - K_2 \cdot H_1)\, dv \tag{S10}$$

Using the Dirac's delta to represent our surface current as volume currents $(J_\mathrm{a}, K_\mathrm{a}) * \delta S$, where δS is surface

differential, the volume integrals involving the adjoint currents become surface integrals, and therefore the Lorentz reciprocity applied to our pairs of current-field becomes:

$$\int_S (J_{\mathrm{a}} \cdot \delta E - K_{\mathrm{a}} \cdot \delta H)\, da = \int_\chi (J_\delta \cdot E_{\mathrm{a}}) dv \tag{S11}$$

Note that the volume integrals involving J_δ is performed only in the region χ, because only there J_δ is non-zero. Furthermore, because of our choice for the adjoint currents, we can express the differential in the figure of merit as:

$$\begin{aligned}
\delta F &= \frac{1}{4\,P} \int_S \left[- (n \times H_{\mathrm{t}}^*) \cdot \delta E + (n \times E_{\mathrm{t}}^*) \cdot \delta H \right] da \\
&= \frac{1}{4\,P} \int_S \left[J_{\mathrm{a}} \cdot \delta E - K_{\mathrm{a}} \cdot \delta H \right] da \\
&= \frac{1}{4\,P} \int_\chi (J_\delta \cdot E_{\mathrm{a}}) dv
\end{aligned} \tag{S12}$$

Using the expression for J_δ and writing $E_{\mathrm{a}} = E_{\mathrm{a},\|} + \frac{1}{\epsilon_2} D_{\mathrm{a},\perp}$(which is exact), we obtain:

$$\delta F = \frac{j\omega\delta\epsilon}{4\,P} \int_\chi \left(E_\| \cdot E_{\mathrm{a},\|} + \frac{1}{\epsilon\,\epsilon\prime} D_\perp \cdot D_{\mathrm{a},\perp} \right) dv \tag{S13}$$

The reciprocity theorem allows us to predict the change in the figure of merit by performing an integration involving quantities directly at the metasurface region, and not on the output surface S. This also means that maximizing this integral in χ directly maximizes our figure of merit. The integration in the volume χ can be mapped to an integration on the surface of the original shape. Say s denotes the arc length along the closed boundary Γ encircling the nanopillar and $u_\perp$ is the normal component of the boundary displacement (Figure S2). The volume element is therefore $dv = u_\perp ds dz$. Assuming the shape only changes in the x,y plane (i.e. all pillars are uniform along the propagation direction z), then:

$$\delta F = \frac{j\omega\delta\epsilon}{4\,P} \int u_\perp \left(E_\| \cdot E_{\mathrm{a},\|} + \frac{1}{\epsilon\,\epsilon\prime} D_\perp \cdot D_{\mathrm{a},\perp} \right) ds dz \tag{S14}$$

and the change in efficiency can be written finally as:

$$\delta\eta = \frac{\omega\delta\epsilon}{2\,P} \int_\Gamma (u_\perp g) ds \tag{S15}$$

where we define a gradient function $g = Re\left[jF^* \int \left(E_\| \cdot E_{\mathrm{a},\|} + \frac{1}{\epsilon\,\epsilon\prime} D_\perp \cdot D_{\mathrm{a},\perp} \right) dz \right]$, which includes an integration along the pillar height. The efficiency change is always positive if we choose $u_\perp = h\ sign\,(\delta\epsilon)\ g$, where h is a scaling factor.

With that, the algorithm is summarized as follows:

- Initialize the structure with a set of known geometrical features (meta-atoms), with any shape. For example, a set of uniform circular pillars or a metasurface previously design with a library approach;
- Compute the forward E and adjoint E_{a} fields;
- With these fields, calculate the gradient function g along the boundary of every pillar;
- Update the boundary shape by displacing every point in its perimeter by $u_{\perp} = h\ sign\left(\delta\epsilon\right)\ g$. Go back to step 2 until the efficiency reaches a local maximum;

The scaling factor h does not alter the deformation function $u_{\perp}$ but simply scales how much the overall shape is deformed. Obviously if h is too large, the approximation for E' and thus for J_{δ} eventually becomes a poor one. There are many ways in which h could be chosen. A simple method is to first calculate h so that the efficiency change is bounded (say no more than 1$\%$ or no less than 0.01$\%$ or any other value). Then, one might impose the maximum of $u_{\perp}$ (say less than 5 nm) and re-scale h. A more general method to deform the boundary is using level-set functions.

1.2 Gradient validation

To validate the gradients obtained using the adjoint formulation, we computed how the first order diffraction efficiency change as the parameters of the geometries are varied. In Figure S3a, the diffraction efficiency is plotted as a function of the width w of a rectangular ridge for both TE (y-pol) and TM (x-pol) polarized light. The derivatives were then numerically calculated and plotted in solid lines in Figure S3c. For comparison, the derivatives obtained directly from the adjoint gradient are shown in circles, in excellent agreement with the exact ones. In Figure S3b, the efficiency of a metasurface that contains two circular pillars is calculated as a function of d_2, diameter of the second pillar (while d_1 is fixed). Again, the derivates computed from the efficiency curves (solid lines) match the one obtained from the adjoint gradient, as shown in Figure S3d.

1.3 Fourier decomposition

It is often desirable to restrict the shape to simple smooth geometries, for example to simplify fabrication. The deformation function $u_{\perp}$ calculated as outlined here may be very complicated, with sharp peaks or valleys. Since we are dealing with a closed boundary, any function can be expanded in terms of an appropriate basis. We chose

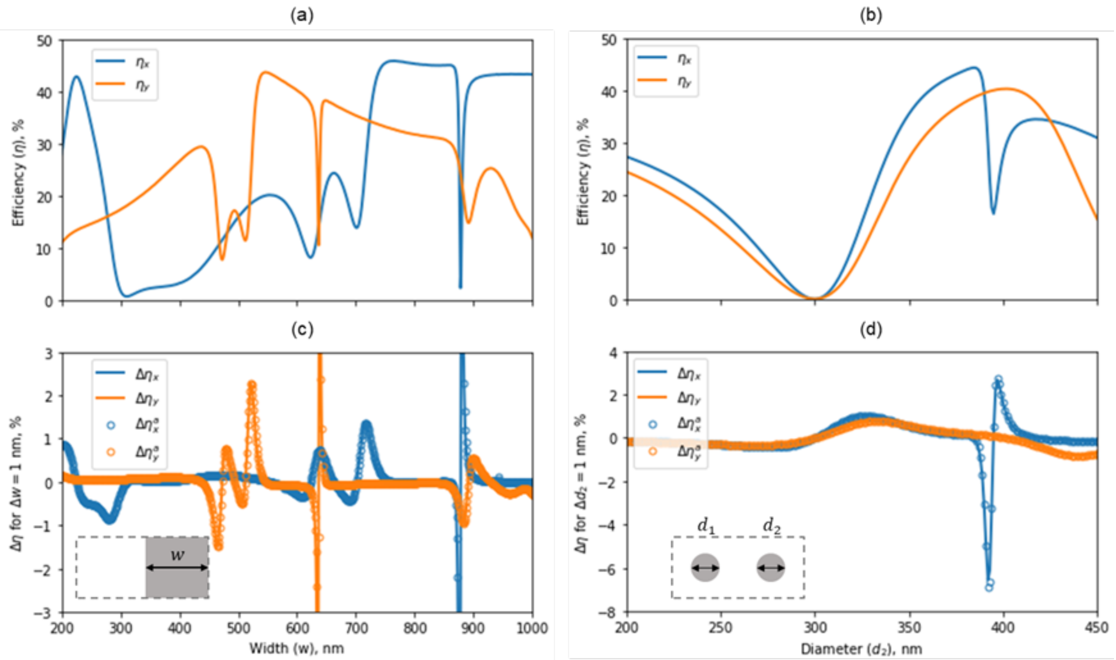

Figure S3: Validation of the boundary gradient calculation. In (a), the efficiency of a metagrating containing only one rectangular nanopillar is numerically calculated as a function of the width w, and in (b) the efficiency of a metasurface with two circular pillars is calculated as a function of the diameter of one of them (d_2). Here, blue and orange represent efficiency for x-polarized (along larger domain direction) and y-polarized light (along smaller domain direction), respectively. From the efficiency, the exact gradient (solid line) is calculated for the two metasurfaces and compared with the gradient obtained from the boundary shift expression (open dots). Top and bottom figures share the same horizontal axis.

Fourier as it easily allows restriction to smooth round structures (the arguments presented here can be generalized to any basis). The gradient function g can be expanded in terms of its Fourier components:

$$g = \frac{a_0}{2} + \sum_{m=1}^{\infty} a_m \cos m\theta + b_m \sin m\theta \tag{S16}$$

where

$$a_m = \frac{1}{\pi}\int_{-\pi}^{\pi} g \cos m\theta d\theta, b_m = \frac{1}{\pi}\int_{-\pi}^{\pi} g \sin m\theta d\theta, \text{ and } \theta = 2\pi\frac{s}{s_\mathrm{t}} \tag{S17}$$

Here, s_t is the total boundary length of a given meta-atom. A circular shape is maintained if we retain only the zero-order term a_0: $u_\perp = h\ sign\,(\delta\epsilon)\ \frac{a_0}{2}$. Retaining first order terms represent slight displacements, second order terms represent elliptical distortions and so on. In general:

$$u_\perp = h\ sign\,(\delta\epsilon) \left(\frac{a_0}{2} + \sum_{m=1}^{\infty} a_m \cos m\theta + b_m \sin m\theta\right) \tag{S18}$$

and therefore, the change in efficiency is:

$$\delta\eta = \frac{\omega\delta\epsilon}{4\,P}\, hs_{\mathrm{t}} sign\left(\delta\epsilon\right)\left[\frac{a_0^2}{2} + \sum_{m=1}^{\infty}\left(a_m^2 + b_m^2\right)\right] \tag{S19}$$

Note that due to the orthogonality of the Fourier basis, each term contributes independently to an increase in efficiency. Therefore, we can choose to retain a finite number of terms. Finally, if we want to optimize for multiple parameters, for example TE and TM polarizations, one way is to define multiple figures of merit, each leading to its own gradient functions:

$$\delta\eta_{\mathrm{TE}} = \frac{\omega\delta\epsilon}{2\,P}\int_{\Gamma} u_{\perp} g_{\mathrm{TE}} ds, \text{ and } \delta\eta_{\mathrm{TM}} = \frac{\omega\delta\epsilon}{2\,P}\int_{\Gamma} u_{\perp} g_{\mathrm{TM}} ds \tag{S20}$$

They can be combined in different ways, for example by using the inverse of efficiency as a weighing function ($w_{\mathrm{TE,TM}} = 1/\eta_{\mathrm{TE,TM}}$) so that the optimization tends to balance well the performance of both polarizations, $g = \left(w_{\mathrm{TM}} g_{\mathrm{TM}} + w_{\mathrm{TE}} g_{\mathrm{TE}}\right) / \left(w_{\mathrm{TM}} + w_{\mathrm{TE}}\right)$. One may also define a single figure of merit as the sum of multiple figures of merit, for example to take into account different wavelengths.

1.4 General figure of merit

The formulation here was based on a specific definition of the figure of merit, expressed as the projection of the output field into the target field, i.e., $\eta = |F|^2$ where $F = \frac{1}{4\,P}\int_S \left(E \times H_{\mathrm{t}}^* + E_{\mathrm{t}}^* \times H\right)\cdot n da$. This can be generalized as follows:

$$F = \int_V f\left(E, H\right) dv \tag{S21}$$

where for convenience, we normalize f so that it has units of inverse of volume. The change in efficiency is then expressed as:

$$\delta F = \int_V \left(\frac{\partial f}{\partial E}\cdot \delta E + \frac{\partial f}{\partial H}\cdot \delta H\right) dv \quad \text{and} \quad \delta F = \frac{1}{4\,P}\int_S \left(J_{\mathrm{a}}\cdot \delta E - K_{\mathrm{a}}\cdot \delta H\right) da \tag{S22}$$

where care must be taken to deal with derivatives with respect to complex vectorial fields. The formulation then follows identically if we choose the adjoint currents as:

$$\left(J_{\mathrm{a}}, K_{\mathrm{a}}\right)\delta S = \left(\frac{\partial f}{\partial E}, -\frac{\partial f}{\partial H}\right) \tag{S23}$$

2 Experimental setup

A schematic of the experimental setup is shown in Figure S4. The linearly polarized output from a Santec TSL-570 tunable laser is collimated and sent through a focusing lens to create a smaller sized beam at the focal plane. A $\lambda/2$ plate is placed after the lens to control the orientation of the linear polarization. The sample is located at the focal plane of the lens, which is set to coincide with the image plane of the camera. A 10x objective and a tube lens are used to image the sample onto the camera which is used for alignment. A power meter is placed at different positions along the beam path to measure input power and diffracted power. The alignment of the system is described in 2.1 and the measurement procedure is outlined in 2.2. Finally, the data processing for comparison with simulation is detailed in 2.3.

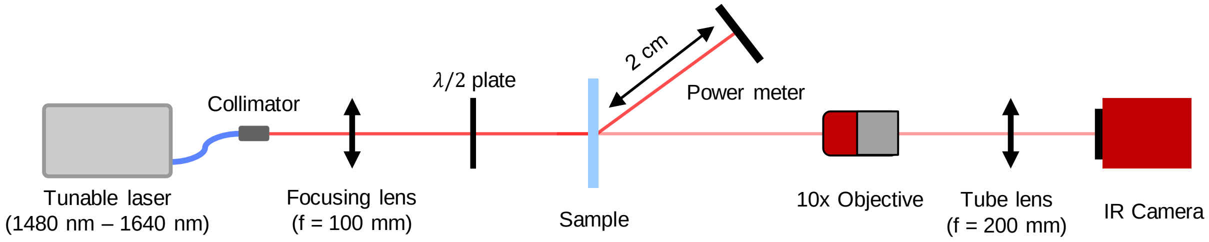

Figure S4: Schematic of the experimental setup used to measure diffraction efficiency.

2.1 Alignment

First, the beam is aligned onto the camera without the sample in the system. Then, the position of the focusing lens is adjusted until the focus of the beam coincides with the image plane of the camera. With a focal length of 100 mm, the focusing lens produces a beam with a diameter of approximately 100 μm at the focus for a wavelength of λ=1550 nm. This beam size is chosen to ensure that the beam is small enough to fit entirely within one of the 250 μm x 250 μm grating patterns. Next, the polarization is set by rotating the $\lambda/2$ plate to minimize the power passing through a linear polarizer that is set to horizontal (x-polarization) or vertical (y-polarization) relative to the table. Then, the sample is placed at the focus of the beam in the image plane of the camera. With the sample positioned so that the beam passes through the bare glass substrate, interference fringes resulting from a tilt of the sample are visible on the camera. The sample is aligned for normal incidence by adjusting the tilt angle until these interference fringes disappear. Finally, the sample is positioned so that the beam passes through one of the grating patterns and is rotated until the height of the diffracted beam matches the height of the input beam.

2.2 Measurement procedure

The input power is measured by placing the power meter in the beam path right before the sample. The power is measured as the tuneable laser is swept from 1480 nm – 1640 nm in steps of 0.1 nm. Then, the beam is centered onto one of the grating patterns with the help of the imaging system. The diffracted power is measured by placing the power meter in the path of the 1st-order diffracted beam, positioned as close as possible to the sample while ensuring that the 0th-order beam is not detected by the sensor. Again, the power is measured while the tuneable laser is swept over the same wavelength range.

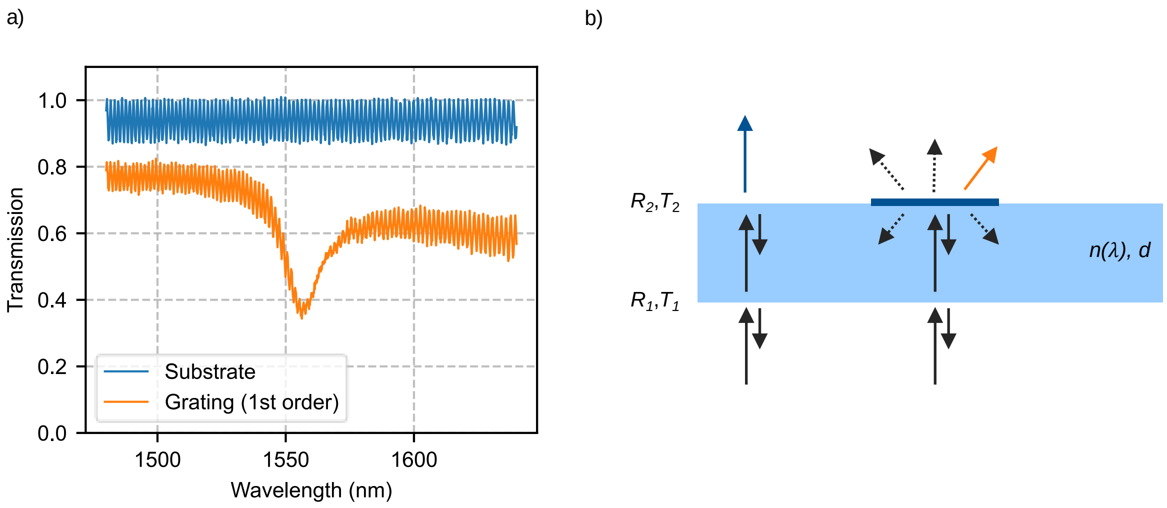

Figure S5: (a) The measured transmission through the bare substrate (blue line) and the 1st-order diffraction of one of the gratings (orange line). (b) Diagram of the Fabry-Perot model for both the bare substrate and one of the grating patterns. The blue- and orange-colored arrows represent where the transmission was measured for the bare substrate and grating, respectively.

2.3 Data processing

In our simulations, the diffraction efficiency of the device is defined relative to the power inside the substrate before interaction with the metasurface. During measurement, however, we can only measure the power before the substrate and use this as our reference. This results in many oscillations in the measured transmission due to interference within the fused silica substrate, as shown in Figure S5a. This can be understood by recognizing that the substrate acts as a Fabry-Perot cavity between the surfaces of the substrate and the 0th-order reflection from the metasurface. A diagram is shown in Figure S5b. The transmission of a Fabry-Perot cavity for normal incidence is given by:

$$T = \frac{T_1 T_2}{1 + R_1 R_2 - 2\sqrt{R_1 R_2} cos(2kn(\lambda)d)} \tag{S24}$$

where $d = 500\ \mu m$ is the thickness of the substrate, $n(\lambda)$ is the wavelength-dependent refractive index of fused silica, $k = 2\pi/\lambda$ is the wavenumber, and $\lambda = 1550\ nm$. In this equation, the parameter T_2 represents the transmission of purely the second interface of the sample, without any interference effects from the first interface. It is the transmission of the sample relative to the power inside the substrate, exactly how we define the diffraction efficiency. With knowledge of the transmission and reflection coefficient for the first interface (T_1 and R_1), the value of T_2 can be extracted from T, which we measure directly. Consider the maximum and minimum values of the Fabry-Perot transmission function. We find

$$T_{\text{max}} = \frac{T_1 T_2}{\left(1 - \sqrt{R_1 R_2}\right)^2} \quad \text{and} \quad T_{\text{min}} = \frac{T_1 T_2}{\left(1 + \sqrt{R_1 R_2}\right)^2} \tag{S25}$$

The values of T_{max} and T_{min} can be extracted from the measurements. R_1 and T_1 are the reflection and transmission coefficient for the back surface of the substrate, with values given by the Fresnel equations. For normal incidence from air to fused silica we have,

$$R_1 = \left| \frac{1 - n(\lambda)}{1 + n(\lambda)} \right|^2 \quad \text{and} \quad T_1 = 1 - R_1 \tag{S26}$$

where $n(\lambda)$ is given by the Sellmeier equation for fused silica. By rearranging the equations for T_{max} and T_{min} we can find T_2 by first solving for the unknown quantity $\sqrt{R_1 R_2}$ using the equations below:

$$\sqrt{R_1 R_2} = \frac{\left(1 - \sqrt{\frac{T_{\text{min}}}{T_{\text{max}}}}\right)}{\left(1 + \sqrt{\frac{T_{\text{min}}}{T_{\text{max}}}}\right)} \quad \text{and} \quad T_2 = \frac{T_{\text{max}} \left(1 - \sqrt{R_1 R_2}\right)^2}{T_1} \tag{S27}$$

To verify this procedure for extracting T_2, we first consider the case of a beam passing through the bare substrate for which T_2 can be readily calculated according to the Fresnel equations. The results are shown in Figure S6.

Using the procedure outlined above for the case of transmission through the bare substrate, we find an average value of $T_{2,\text{ measured}} = 0.9670079$ which agrees very well with the value given by the Fresnel equations $T_{2,\text{ Fresnel}} = 0.9670084$. The same processing is used for the 1st-order transmission measurements of the meta-gratings to calculate the diffraction efficiency. An example is shown in Figure S7.

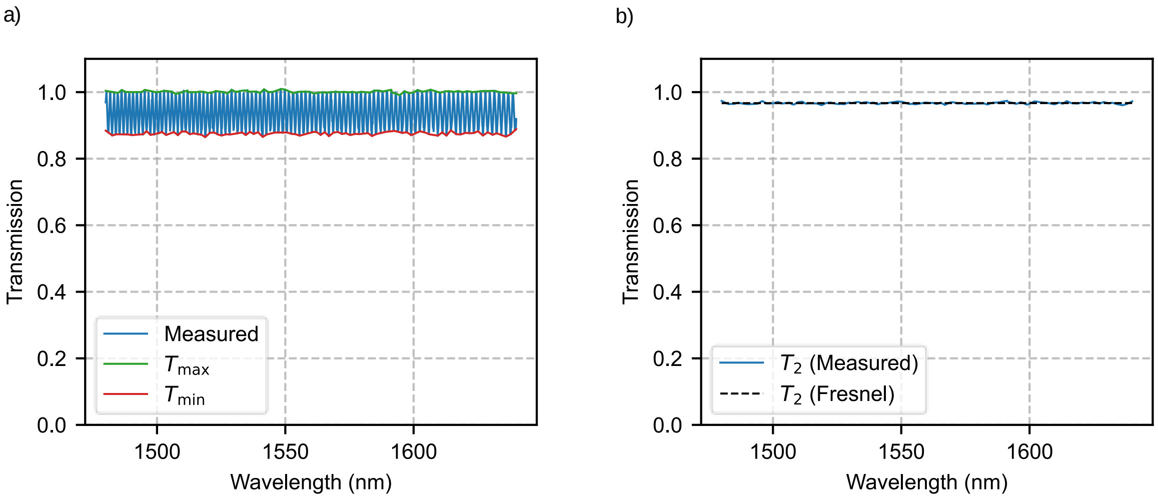

Figure S6: (a) The values of $T_{\max}$ (green curve) and $T_{\min}$ (red curve) are interpolated from the measured transmission (blue curve) through bare substrate. (b) The extracted value of T_2 (blue curve) is compared with the exact value given by the Fresnel equations (dashed black line).

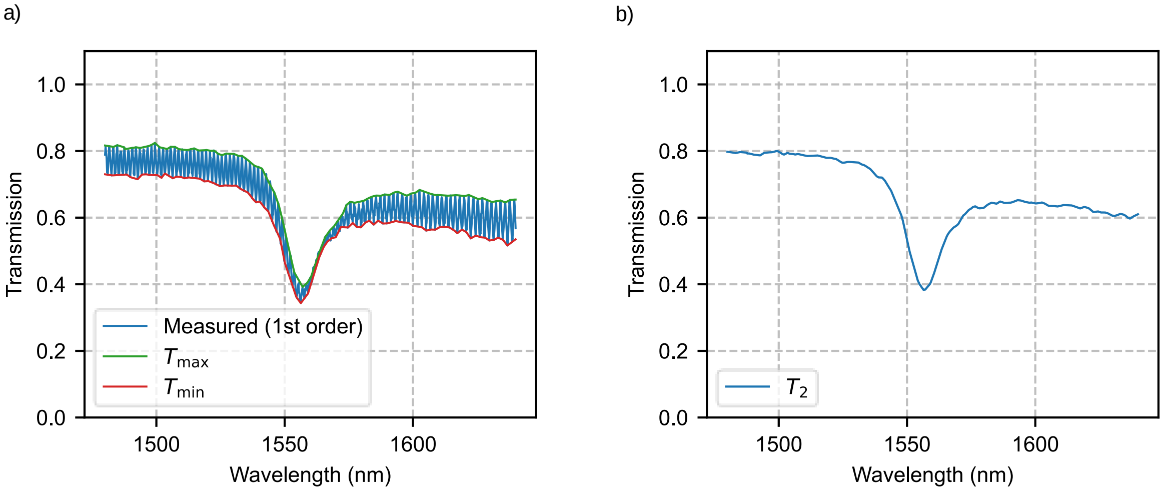

Figure S7: (a) The values of $T_{\max}$ (green curve) and $T_{\min}$ (red curve) are interpolated from the measured 1st-order transmission (blue curve) of a meta-grating. (b) The extracted value of T_2 (blue curve), defining the measured diffraction efficiency.

References

[1] Steven G Johnson, Mihai Ibanescu, MA Skorobogatiy, Ori Weisberg, JD Joannopoulos, and Yoel Fink. “Perturbation theory for Maxwell’s equations with shifting material boundaries”. In: *Physical Review E* 65.6 (2002), p. 066611.

[2] K M Chen. “A mathematical formulation of the equivalence principle”. In: *IEEE Transactions on Microwave Theory and Techniques* 37.10 (1989), pp. 1576–1581.

[3] Lev Davidovich Landau, John Stewart Bell, MJ Kearsley, LP Pitaevskii, EM Lifshitz, and JB Sykes. *Electrodynamics of continuous media*. Vol. 8. Elsevier, 2013.
